# Efficacy and tolerability of gefitinib in pretreated elderly patients with advanced non-small-cell lung cancer (NSCLC)

**DOI:** 10.1038/sj.bjc.6601470

**Published:** 2004-01-06

**Authors:** F Cappuzzo, S Bartolini, G L Ceresoli, S Tamberi, A Spreafico, L Lombardo, V Gregorc, L Toschi, C Calandri, E Villa, L Crinò

**Affiliations:** 1 Bellaria Hospital, Division of Medical Oncology, Via Altura 3, Bologna 40139, Italy; 2 Division of Radiochemotherapy, Scientific Institute University Hospital, San Raffaele, Milano, Italy; 3 Faenza Hospital, Division of Medical Oncology, Faenza, Italy

**Keywords:** thyrosine kinase inhibitor, elderly, non-small-cell lung cancer

## Abstract

The activity and toxicity profile of gefitinib in non-small cell lung cancer (NSCLC) patients aged 70 years or older has been only partially evaluated. The aim of this study was to evaluate the response rate and safety of gefitinib in elderly NSCLC patients. Elderly NSCLC patients pretreated with chemotherapy and with at least one measurable lesion received gefitinib at the daily dose of 250 mg until disease progression, unacceptable toxicity or refusal. From August 2001 to May 2003, 40 consecutive elderly patients have been enrolled onto the study in three Italian institutions. We observed one complete (2.5%) and one partial response (2.5%), 18 disease stabilisations (NC: 45%) lasting at least 2 months, including six patients (15%) who had disease stabilisation of 6 months or longer, for an overall disease control rate of 50% (95% CI: 34.5–65.5%). The median duration of response was 4.4 months (range 1.7–9.2). The side effects were generally mild and consisted of diarrhoea and skin toxicity. Grade 1–2 diarrhoea occurred in 23.6%, and one patient experienced grade 4 diarrhoea, requiring hospitalisation. Grade 1–2 skin toxicity, including rash, pruritus, dry skin, and acne, occurred in 20 patients (52.6%). Gefitinib is safe and well tolerated in elderly pretreated NSCLC patients. The disease-control rate achieved suggests that this drug could represent a valid option in the management of this unfavourable subgroup of patients.

Non-small-cell lung cancer (NSCLC) is the most lethal malignant tumour in the United States with an estimated 169 500 new patients diagnosed and 157 400 deaths in 2001. Approximately 30% of those diagnosed are patients aged 70 years or older (Hutchins *et al*, 1999; Greenlee *et al*, 2001). Treatment of elderly NSCLC patients represents a challenge in clinical practice, because these patients are not eligible for aggressive therapies due to the age-related reduction of the functional reserve of many organs and comorbidities (Balducci *et al*, 2000). Recent data suggest that platinum-based chemotherapy can be safely proposed to the elderly (Langer *et al*, 2002), but so far mainly single-agent chemotherapy with vinorelbine or gemcitabine has been considered as the standard treatment for advanced disease (Gridelli *et al*, 2003). Although elderly patients have not been excluded from randomised phase III trials evaluating the activity of second-line chemotherapy (Shepherd *et al*, 2000; Hanna *et al*, 2003), only very selected patients with good performance status are candidates to salvage chemotherapy in everyday clinical practice.

Since its identification, the epidermal growth factor receptor (EGFR) has emerged as a significant factor in the development and growth of many types of cancers. It is now accepted that the EGFR signal transduction network plays an important role in multiple tumorigenic processes, contributing to cancer-cell proliferation, angiogenesis, and metastasis, as well as protection from apoptosis (Huang and Harari, 1999). Since the majority of NSCLC expresses EGFR, this represents a rational target for novel agents that can block cancer cell growth by interfering with this mitogenic signalling pathway. Gefitinib (ZD1839, IRESSA, Astrazeneca, London, UK) is an orally active, selective EGFR tyrosine-kinase inhibitor (TKI) that blocks signal transduction pathways implicated in the proliferation and survival of cancer cells (Rusch *et al*, 1993; Salomon *et al*, 1995). The 11% response rate observed in phase I trials (Kris *et al*, 2000) led to two large phase II studies evaluating the activity and tolerability of two different gefitinib doses. In the first trial (IDEAL 1), 210 pretreated NSCLC patients were randomised to ZD 1839 250 or 500 mg daily dose (Fukuoka *et al*, 2003). This trial showed an 18% response rate with symptomatic improvement in 40% of patients, without any significant difference between the two dose levels. In the US trial (IDEAL 2), 216 NSCLC patients, who had failed in two or more prior chemotherapy regimens containing platinum and docetaxel, were randomly assigned to ZD 1839 250 or 500 mg daily. This trial confirmed that ZD 1839 is active in heavily pretreated NSCLC patients, with a response rate of 11.8% and symptom improvement in 43% of patients in the 250 mg arm (Kris *et al*, 2000). Although the age of 70 years or older was not considered as an exclusion criteria, in all these studies, the safety and activity of gefitinib in the elderly population has not been directly addressed. At the daily dose of 250 mg, skin toxicity and diarrhoea are the main gefitinib side effects, occurring in 46.6 and 39.8% of cases, respectively (Fukuoka *et al*, 2003). In the elderly, the risk of diarrhoea is of particular importance due to dehydration and electrolyte imbalance that can even be life threatening. Moreover, although gefitinib is considered a very well-tolerated drug, recent findings suggest that in some patients (1–2% in Japan) gefitinib has the potential of causing acute interstitial pneumonia, and this side effect is more common in patients with comorbidities or those who received previous mediastinal radiotherapy (Inoue *et al*, 2003). Based on these considerations, we decided to assess the safety and activity of gefitinib in pretreated elderly patients with advanced NSCLC. To conduct this trial, we used gefitinib provided by Astrazeneca within the expanded access program.

## PATIENTS AND METHODS

### Patient selection

The eligibility criteria were: histologic or cytologic confirmation of locally advanced or metastatic NSCLC; stage III or stage IV disease not amenable to surgery or radiotherapy at the study entry; recurrent or refractory disease following at least one previous chemotherapy regimen; evidence of disease progression at the time of study entry; at least one bidimensionally measurable or radiographically assessable lesion; age of 70 years or older; ECOG performance status (PS) of 0–2; life expectancy of at least 12 weeks; and white blood cell count *≥*3.5 × 10^9^ 1^−l^, platelets *≥*100 × 10^9^ 1^−l^, haemoglobin *≥*9 g dl^−1^, and absolute granulocyte count (AGC) >2.0 × 10^9^ 1^−l^; bilirubin <1.5-fold the upper limit of normal (ULN). Evaluation of creatinine level was not required for trial inclusion. Patients with stable brain metastases were eligible. Written informed consent was obtained from each patient before entering the study. The study was conducted after the approval of the appropriate ethical review boards. Recommendations of the Declaration of Helsinki for biomedical research involving human subjects were also followed.

### Study design and treatment

In this study, consecutive elderly NSCLC patients received gefitinib at the daily dose of 250 mg given until disease progression, unacceptable toxicity or refusal. Baseline evaluation included a complete history and physical examination, a complete blood cell count and serum chemistry analysis, urinalysis, an ECG, chest X-ray, and a total body computed tomography scan. Other imaging modalities, such as magnetic resonance imaging and bone scintigraphy, were performed according to specific clinical indications. All baseline imaging procedures were performed within 4 weeks before the study entry. After trial inclusion, toxicity and disease-related symptom assessment were performed every 28 days. Toxic effects were assessed according to the NCI CTC (Common toxicity criteria: National Institute of Health, Cancer Therapy Evaluation Program, Division of Cancer Treatment, 1993). Symptom assessment was carried out by the physician, and no questionnaire was used. The side effects and safety were evaluated clinically and through the assessment of serum creatinine, electrolytes, alkaline phosphatase, bilirubin, AST, ALT, calcium, magnesium, and protein levels. Chest X-ray was repeated after 1 month of gefitinib therapy and thereafter in the case of lung disease suspicion. Patients were evaluated for response according to the RECIST Criteria (Therasse, 2000). Tumour response was assessed by computer tomography (CT) scan every 2 months, with a confirmatory evaluation to be repeated in the responding patients at least 4 weeks after the initial determination of response.

### Statistical considerations

The number of 40 patients to enroll onto the study was calculated according to the method by Gehan (1961). This was to ensure that the drug had a less than 20% disease control rate, and that the study could be terminated with a maximal error of 5% in estimation of the true response rate. Every patient included in the trial has been considered evaluable (intent to treat analysis). Stable disease was measured from the start of the treatment until the criteria for disease progression was met. The duration of overall response was measured from the time that criteria were met for complete response or partial response, until the first date that recurrent or progressive disease was documented.

## RESULTS

### Patient characteristics

From August 2001 to May 2003, 40 consecutive elderly patients were treated with a daily dose of 250 mg of ZD 1839 in three Italian institutions. Of these, 40 patients were evaluable for efficacy, and 38 patients were evaluable for safety. In two cases, safety data were not recorded on the patient file. Characteristics of the eligible patients are listed in Table 1

**Table 1:** Patient characteristics

**Total enrolled: 40**	***N***	**%**
*Sex*		
Male	33	82.5
Female	7	17.5

*Age (year)*		
Median	74	
Range	70–88	

*Stage*		
IIIB	11	27.5
IV	29	72.5

*Histology*		
Squamous cell carcinoma	14	35
Adenocarcinoma	18	45
Bronchiolar–alveolar carcinoma	4	10
Undifferentiated NSCLC	4	10

*Site of metastases*		
Lung	23	
Liver	3	
Bone	7	
Brain	2	
Pleura	4	
Adrenal	2	
Skin	2	

*Number of metastatic sites*		
0	11	27.5
1	19	47.5
2	6	15
3+	4	10

*ECOG performance status*		
0	10	25
1	27	67.5
2	3	7.5
		
Symptoms at study entry	20	50

*Previous chemotherapy lines*		
1	21	52.5
2	16	40
3+	3	7.5
Pretreated with platinum	19	47.5

. The majority of patients were male (82.5%), with a median age of 74 years (range 70–88), and with an ECOG PS of 0, 1, and 2 in 10, 27, and 3 patients, respectively. Histology was adenocarcinoma in 45%, bronchiolar–alveolar in 10%, squamous-cell carcinoma in 35%, and undifferentiated carcinoma in 10% of cases. Lung represented the main metastatic site (23 cases), and two patients had brain metastases. The majority of patients had only one metastatic site (47.5%), and only 10% of patients had three or more organs involved by the disease. All patients have been pretreated with chemotherapy. In all, 19 patients (47.5%) received platinum-based chemotherapy, while 21 patients were treated with non-platinum compounds. A total of 21 patients (52.5%) received gefitinib as second-line therapy, and only three patients (7.5%) received the experimental drug after the failure of three chemotherapy lines. At study entry, 29 patients (72.5%) were stage IV, and 11 (27.5%) were stage III, including three patients with pleural effusion. Four patients with stage III disease and without pleural effusion had been pretreated with mediastinal radiotherapy completed at least 4 months before study entry, and all had evidence of disease progression. In total, 28 patients were current smokers, seven former smokers, and five no smokers. The most frequent comorbidity was cardiovascular, occurring in 28 patients (70%). The vast majority of patients were on treatment for hypertension (57%), or had a history of coronary artery disease or stroke (25%). Respiratory symptoms were reported in eight patients (20%), mainly because of chronic obstructive pulmonary disease. Although specific renal diseases were not reported, 10 patients (25%) presented elevated creatinine levels at study entry (Table 2

**Table 2:** Baseline comorbidities

	*N*	**%**
Total patients	40	100

*N comorbidity*		
0	12	
1	10	
2	8	
>2	10	

*Type*		
Cardiovascular	39	
Respiratory	8	
Neurologic	2	
Diabetes	3	
Urinary	5	

*Creatinine level*		
Median value (mg dl^−1^)	1.04	
Range (mg dl^−1^)	0.59–2.72	
<1.2 mg dl^−1^	23	
>1.2 mg dl^−1^	10	
Not available	7	

).

### Response to therapy

Among the 40 patients enrolled, we observed one complete (CR: 2.5%) and one partial response (PR: 2.5%), for an overall response rate of 5%. A total of 18 patients (45%) had disease stabilisation lasting at least 2 months, including six patients (15%) who had a disease stabilisation of 6 months or longer, for an overall disease-control rate of 50% (95% CI: 34.5–65.5%). No difference in response rate was observed in platinum-naïve *vs* platinum-pretreated patients. Partial response was observed in a patient previously treated with platinum-based chemotherapy. Complete response was observed in an 83-year-old platinum-naïve patient, who experienced grade 3 skin toxicity that caused temporary therapy withdrawal. In this patient, CT scan performed after 3 months of gefitinib therapy showed PR, while the CT scan performed after 10 months of therapy demonstrated complete disappearance of the disease. The median duration of response was 4.4 months (range 1.7–9.2). So far, 25 patients are still alive and eight patients are on treatment, including the two responding patients (6.6+ and 7+ months). The median time to progression and overall survival time were 3 and 5 months, respectively.

### Toxicity and symptom outcome

The side effects were generally mild and consisted of diarrhoea and skin toxicity. Grade 1–2 diarrhoea occurred in nine patients (23.6%), and one patient experienced grade 4 diarrhoea, requiring hospitalisation. This patient refused to continue therapy, despite radiological evidence of disease stabilisation. In all the other patients, diarrhoea resolved while on treatment, without dose reduction or suspension. Grade 1–2 skin disorders, including rash, pruritus, dry skin, and acne, occurred in 20 patients (52.6%). In these patients, skin toxicity did not require any change of therapy. Two patients experienced grade 3 skin toxicity. In these patients, this side effect required temporary drug discontinuation. No one experienced interstitial lung disease-type event during the study. Patients had no deterioration in hepatic function, and no clinically significant deterioration in renal function was observed during the trial, even in patients who entered the trial with mild or moderate renal impairment. No clinically significant changes in haematologic parameters were observed during the trial; most patients experienced no changes from baseline in CTC grade for haemoglobin, platelets, or WBC values. Grade 1 or 2 drug-related ophthalmic side effects not requiring any therapy modification were observed in seven patients (18.4%). At study entry, 20 patients presented disease-related symptoms mainly consisting of dyspnea (17.5%) and asthenia (15%). After 1 month of gefitinib therapy, disease-related symptoms improved in 35%, remained stable in 25%, and got worse in 40% of cases (Table 3

**Table 3:** Symptom outcome

		**Outcome after 1 month**		
**Baseline symptoms**	***N* (%)**	**Better (%)**	**Stable (%)**	**Worse (%)**
Total patients	40 (100)	17.5	42.5	40.0
Total with symptoms	20 (50)	35.0	25.0	40.0
Dyspnea	7 (17.5)	28.6	28.6	42.8
Asthenia	6 (15.0)	16.7	50.0	33.3
Emoftoe	2 (5.0)	100	0	0
Bone pain	5 (12.5)	20.0	20.0	60.0
Cough	2 (5.0)	100	0	0

). Symptomatic improvement occurred in five patients experiencing disease stabilisation, and also in two patients with progressive disease.

## DISCUSSION

This study shows that gefitinib, at the daily dose of 250 mg, can be safely administered in elderly NSCLC patients. The overall response rate was 5, and 45% of patients had disease stabilisation. All patients had advanced NSCLC relapsing or progressing after first-line chemotherapy. At the present time, the only FDA-approved drug for second-line therapy is docetaxel, with a response rate of almost 6% in randomised phase III trials (Fossella *et al*, 2000; Shepherd *et al*, 2000). Although elderly patients have not been excluded from second-line chemotherapy trials (Shepherd *et al*, 2000; Hanna *et al*, 2003), in clinical practice, a second option is generally offered only to well-selected cases, with good performance status and without significant comorbidities. Gefitinib is a TKI that was demonstrated to be active in pretreated NSCLC patients (Kris *et al*, 2000). The activity and tolerability of gefitinib as a single agent has been evaluated in two large phase II studies (Kris *et al*, 2000; Fukuoka *et al*, 2003), and also in smaller studies with unselected pretreated NSCLC patients enrolled in the ZD1839 Expanded Access Programme (Janne *et al*, 2002; Ruckdeschel *et al*, 2002; Argiris *et al*, 2003; Kommareddy *et al*, 2003; Pallis *et al*, 2003). All these trials showed that gefitinib is a valid option for pretreated NSCLC patients, with response rates ranging from 5 to 18%, and with a consistent portion of patients experiencing disease stabilisation. Although elderly patients were also enrolled in all these trials, the activity of gefitinib in this subgroup has not been addressed. The response rate (CR +PR) observed in our trial was only 5%, lesser than that reported in the IDEAL 1 and 2 trials, but not different from that reported in other compassionate use studies (Janne *et al*, 2002; Ruckdeschel *et al*, 2002; Argiris *et al*, 2003; Copin *et al*, 2003; Kommareddy *et al*, 2003; Pallis *et al*, 2003). Moreover, in our study, disease stabilisation has been documented in 45% of patients, and, importantly, 15% had disease stabilisation lasting at least 6 months. This finding is interesting because prolonged disease stabilisation has been obtained in patients with evidence of progressive disease when gefitinib therapy was started. These data suggest that disease stabilisation has been caused by the drug and it is not related to the natural history of a slow-growing tumour. The rate of disease control was comparable to that obtained in the IDEAL 1 trial, in which it was 54.4% (Kris *et al*, 2002). With respect to safety, therapy was well tolerated and side effects were mild, consisting mainly of diarrhoea and skin reactions. Before starting the study, we were weary about the risk of inducing diarrhoea in this particular group of patients. The overall incidence of diarrhoea was 26.2%, and it was generally mild. Only two patients had grade 2 diarrhoea, requiring loperamide treatment. The only patient who experienced grade 4 diarrhoea was hospitalised, discontinued the drug, and received rehydration therapy plus loperamide, with disappearance of symptoms in 1 day. Skin toxicity occurred in 67.8% of cases and did not require any specific therapy. Only patients with grade 2 skin toxicity associated with pruritus required systemic administration of steroids and/or antihistaminics, with benefit and no drug suspension. In the two patients who experienced grade 3 skin toxicity, steroids and antihistaminics were ineffective and therapy was temporarily discontinued. Monitoring of renal and hepatic function did not reveal any abnormality. The risk of lung toxicity, such as interstitial lung-disease-type events, has been evaluated by chest X-ray performed after 1 month of gefitinib therapy, and also in all cases of worsening or new-onset dyspnea. No patient experienced lung toxicity, and, in the three patients in whom there was worsening dyspnea, this was mainly due to disease progression. The side effects reported in our study are similar to those reported in other trials in which diarrhoea and skin toxicity occurred in about 40 and 50% of cases (Janne *et al*, 2002; Ruckdeschel *et al*, 2002; Argiris *et al*, 2003; Copin *et al*, 2003; Fukuoka *et al*, 2003; Kommareddy *et al*, 2003; Pallis *et al*, 2003). Symptom assessment was not the main end point of our study, and for that reason no questionnaire has been used. Symptoms were evaluated carefully by the physician at each visit, and symptomatic improvement was observed in 35% of patients after 1 month of gefitinib therapy, mainly in those responding to gefitinib therapy, but also in two nonresponding patients. Although a comparison is not possible due to the different evaluation method, our results are similar to those achieved in the IDEAL 1 study, in which symptomatic improvement occurred in 40.3% of patients treated with the 250 mg gefitinib daily dose. In conclusion, this study suggests that gefitinib is safe and well tolerated in elderly pretreated NSCLC patients. Although the response rate is not impressive, the disease-control rate achieved leads to the conclusion that this drug could represent a valid option in the management of this unfavourable subgroup of patients. Further studies comparing gefitinib with chemotherapy or with best supportive care are needed to better define the role of this compound in the elderly.
